# COMMD3 loss drives invasive breast cancer growth by modulating copper homeostasis

**DOI:** 10.1186/s13046-023-02663-8

**Published:** 2023-04-18

**Authors:** Janelle L. Hancock, Murugan Kalimutho, Jasmin Straube, Malcolm Lim, Irma Gresshoff, Jodi M. Saunus, Jason S. Lee, Sunil R. Lakhani, Kaylene J. Simpson, Ashley I. Bush, Robin L. Anderson, Kum Kum Khanna

**Affiliations:** 1 QIMR Berghofer Medical Research Institute, 300 Herston Road, Herston, QLD 4006 Australia; 2 The University of Queensland Faculty of Medicine, UQ Centre for Clinical Research and Anatomical Pathology, Pathology Queensland, Herston, QLD 4029 Australia; 3 Mater Research Institute-The University of Queensland, Translational Research Institute, Woolloongabba, QLD 4102 Australia; 4 Victorian Centre for Functional Genomics, Peter MacCallum Cancer Centre, Melbourne, VIC 3010 Australia; 5 Sir Peter MacCallum Department of Oncology and the Department of Biochemistry and Pharmacology, University of Melbourne, Parkville, VIC 3052 Australia; 6 Florey Institute of Neuroscience and Mental Health, Parkville, VIC 3052 Australia; 7 Olivia Newton-John Cancer Research Institute, Heidelberg, VIC 3084 Australia; 8 School of Cancer Medicine, La Trobe University, Bundoora, VIC 3086 Australia

**Keywords:** Breast cancer, Tumour suppressor, 3D screen, COMMD3, Copper signalling

## Abstract

**Background:**

Despite overall improvement in breast cancer patient outcomes from earlier diagnosis and personalised treatment approaches, some patients continue to experience recurrence and incurable metastases. It is therefore imperative to understand the molecular changes that allow transition from a non-aggressive state to a more aggressive phenotype*.* This transition is governed by a number of factors.

**Methods:**

As crosstalk with extracellular matrix (ECM) is critical for tumour cell growth and survival, we applied high throughput shRNA screening on a validated ‘3D *on-top* cellular assay’ to identify novel growth suppressive mechanisms.

**Results:**

A number of novel candidate genes were identified. We focused on *COMMD3*, a previously poorly characterised gene that suppressed invasive growth of ER + breast cancer cells in the cellular assay. Analysis of published expression data suggested that *COMMD3* is normally expressed in the mammary ducts and lobules, that expression is lost in some tumours and that loss is associated with lower survival probability. We performed immunohistochemical analysis of an independent tumour cohort to investigate relationships between COMMD3 protein expression, phenotypic markers and disease-specific survival. This revealed an association between COMMD3 loss and shorter survival in hormone-dependent breast cancers and in particularly luminal-A-like tumours (ER^+^/Ki67-low; 10-year survival probability 0.83 *vs.* 0.73 for COMMD3-positive and -negative cases, respectively). Expression of COMMD3 in luminal-A-like tumours was directly associated with markers of luminal differentiation: c-KIT, ELF5, androgen receptor and tubule formation (the extent of normal glandular architecture; *p* < 0.05). Consistent with this, depletion of *COMMD3* induced invasive spheroid growth in ER + breast cancer cell lines in vitro, while *Commd3* depletion in the relatively indolent 4T07 TNBC mouse cell line promoted tumour expansion in syngeneic Balb/c hosts. Notably, RNA sequencing revealed a role for COMMD3 in copper signalling, via regulation of the Na^+^/K^+^-ATPase subunit, *ATP1B1*. Treatment of COMMD3-depleted cells with the copper chelator, tetrathiomolybdate, significantly reduced invasive spheroid growth via induction of apoptosis.

**Conclusion:**

Overall, we found that COMMD3 loss promoted aggressive behaviour in breast cancer cells.

**Supplementary Information:**

The online version contains supplementary material available at 10.1186/s13046-023-02663-8.

## Background

Breast cancer is the most frequently diagnosed cancer globally, and the leading cause of cancer-related death amongst women [1, 2]. The recent identification of well-defined molecular subtypes, driver genetic alterations and signaling pathways makes breast cancer one of the few tumour types in which molecular classification has been used successfully for the design of individualised therapies. Early detection and improvements in therapy are increasing the proportion of patients who survive beyond five years after diagnosis [3]. However, systemic therapies give only a small improvement in survival, and mortality rates have remained poor for ~ 20% of patients with aggressive disease [2]. This indicates that systemic chemotherapies are not effectively targeting residual disease. Metastatic breast cancer may emerge from latent tumour cells that remain dormant at disseminated sites for many years [4]. The mechanism regulating the switch from dormancy to proliferative and invasive growth in vivo is not fully understood. Therefore, there is a need for improved mechanistic understanding to drive better therapeutic outcomes in this setting.

Breast cancer is well defined through five major gene expression profile-based (PAM50) subtypes [5, 6]. HER2 + and TNBC subtypes have the worst prognosis overall due to inherent metastatic and drug-resistant traits [7, 8]. Multiple factors are thought to drive these phenotypes in breast cancer [9, 10]. A classic example is HER2 gene amplification – a determinant of aggressive behaviour that is also therapeutically targetable [11–13]. On the other hand, TNBC, which is mainly driven through dysregulation of cell-cycle related genes (e.g., *TP53* mutations), is characterized by a higher mutation burden affecting a wide array of genes, each at low frequency in the patient population [8, 11, 12]. Although the roles of key driver genes are well documented through in vitro and in vivo studies [14], other regulators are also likely to contribute to aggressive breast cancer and render cells resistant to current treatments.

Various genetic and epigenetic modifications, as well as modulation of tumour milieu, have been implicated in acquisition of the invasive state [10]. However, the molecular mechanisms explaining these processes is largely impeded by lack of suitable in vitro model systems. Many large-scale 2D shRNA and siRNA screens have been completed to identify regulators of breast cancer invasiveness and subtype-specific gene dependency [15–18], but such approaches likely under-report mechanisms that underpin proliferation and invasion in a 3D structure. Tumours are a complex milieu of cancer cells with genetic and transcriptional diversity, infiltrating immune cells, fibroblasts, vascular and lymphatic cells, all embedded in and contributing to a protein-rich extracellular matrix (ECM). There is a dynamic reciprocity between cancer and its microenvironment that underpins cancer behaviour and response to therapy [19]. In particular, cancer cell driven matrix remodelling is a key mediator in the establishment of the premetastatic niche. The disseminated estrogen-receptor positive (ER +) breast cancer cells can survive at a secondary site for a long-period of time (often years) before developing into overt metastasis. There is need to consider the environmental factors when seeking to identify novel regulators that are responsible for the invasive phenotype and aggressive behaviour in breast cancer [20].

As a compromise between standard 2D culture and the use of in vivo models in mice with the full complement of host cell microenvironment, we have used a 3D culture model in which the cells are grown on a matrix that resembles the extracellular matrix surrounding tumours in mice or patients. To distinguish novel regulators of the invasive phenotype, the cells in the 3D on-top assay were cultured in low serum (2% FCS) conditions, more similar to the environment of tumours in vivo, where cells are bathed in plasma, not serum. Under these conditions, growth of less aggressive breast cancer lines is suppressed [21]. We utilised two different pooled shRNA libraries (targeting polarity or kinome genes) to identify genes that could promote growth of these less aggressive cells. We identified known tumour suppressors, including CEBPA and novel candidates such as COMMD3. Low expression of COMMD3 was associated with poor outcome in patients treated with chemotherapy, regardless of their subtype. Depletion of COMMD3 in non-aggressive breast cancer cells promoted an invasive phenotype.

## Methods

### Reagents

The copper chelator Tetrathiomolybdate (TM) was purchased from Sigma-Aldrich. Lipofectamine® 3000 Reagents were purchased from Life Technologies, Carlsbad (CA, USA). Matrigel™ Basement Membrane Matrix was obtained from BD Biosciences.

### Analysis of COMMD3 mRNA expression in breast cancer

KMPlotter (http://kmplot.com) was used for breast cancer-specific survival analysis [22]. cBioPortal (http://www.cbioportal.org) was used to generate data related to mRNA expression [23, 24].

### Analysis of COMMD3 protein expression in breast cancer

Immunohistochemistry (IHC) analysis of COMMD3 was performed on a consecutive series of primary breast tumours, with clinical annotation including > 20 years’ survival follow-up data. Patients in this cohort were treated at multiple centres across Queensland, Australia, between 1987 and 1993. Treatment information for these historic cases is not available, but the vast majority (if not all) tumour samples can be considered unaffected by systemic therapy as neoadjuvant treatment was rare in Australia at the time. Thirty-five percent of the patients died from metastatic breast cancer (median survival 4.4 years; vs 20.7 years for the remaining 65% of the cohort).

Tumours sampled in tissue microarrays (TMAs) were subjected to IHC analysis for COMMD3. Briefly, 4 μm TMA sections were heat-retrieved in citrate buffer (0.01 M, pH 6.0) using a decloaking chamber and then stained with anti-COMMD3 (Sigma Aldrich; HPA036584 1:200). The MACH 1 Universal HRP-Polymer Detection Kit was used for detection. Stained sections were counterstained with hematoxylin, mounted and scanned at 40 × magnification on an Aperio AT Turbo slide scanner (Leica Biosystems). De-identified digital TMA core images were scored by one assessor and reviewed by a second (maximum score of duplicate cores from each tumour were used). Analysis included cross-referencing to clinicopathologic parameters that were assessed for this cohort previously, including Ki67, ELF5, AR and cKIT [25–29]. Ethical approval for this study was obtained from human research ethics committees of the Royal Brisbane & Women’s Hospital (RBWH; 2,005,000,785) and The University of Queensland (HREC/2005/022).

### Antibodies

Antibodies used in this study were: COMMD3 (Sigma Aldrich; HPA036584), Lamin A/C (CST; 2032 T), GAPDH (TACS; 22,750-PC-100), PARP (CST; 9542S), ATP7A (GeneTex; GTX101333), and VCP (GeneTex; GTX113030).

### Cell culture

The breast cancer cell lines, apart from 4T07, 66cl4 and 4T1.2, were purchased from the American Type Culture Collection (ATCC), cultured and maintained as per ATCC recommendations and as described previously [30]. 4T07 cells with expression of thymidine kinase, GFP and luciferase were generated for in vivo studies (4T07-TGL). All the cell lines were tested for Mycoplasma infection and human cell lines were authenticated using short tandem repeat (STR) profiling by scientific services at QIMR Berghofer Medical Research Institute.

### ShRNA library transfection and Sequencing

The target genes for the kinome library (903 targets;kinases and kinase-like) and the polarity library (1526 targets) were selected by the Victorian Centre for Functional Genomics (Peter MacCallum Cancer Centre). shRNAs specific to target genes (2–4 hairpins per gene) cloned in the pGIPz shRNAmir30 vector were provided by Open Biosystems in 96 well format as glycerol stocks. DNA was extracted from individual wells, pooled libraries were generated and high titre lentiviral particles were produced in HEK293T cells using Polyethyleneimine (PEI) (for 1 µg DNA, 5µL of 1 mg/ml PEI was used). T-47D cells were transduced with lentiviral particles at an MOI of < 0.5 to minimize chances for multiple hairpin integrations per cell. Cells were then subjected to puromycin selection for 3 days to enrich for transduced cells. For each library, transduced T-47D cells (1.5 × 10^6^) were plated on a growth area of T75 flask of GFR Matrigel overlaid with DMEM 2% FCS as described previously [20]. Day zero samples taken prior to seeding were retained and stored frozen at − 80 °C for subsequent genomic DNA extraction. Day 14 acini were collected by dissolving the Matrigel in ice cold versene and pelleting the cells. Genomic DNA was prepared from day zero and day 14 samples for amplification, sequencing (using pGIPz sequencing primers, X76-pGIPZ: ACGTCGAGGTGCCCGAAGGA; M100-pGIPZ:AAGCAGCGTATCCACATAGCGT) and quantification of shRNA sequences as previously described [31]. The abundance of hairpins before and after acini culture was compared to detect enrichment as described below.

### Data normalisation and ratio detection

The formula below was used to generate a ratio of enrichment score:

(Hairpin counts at day 14 of given shRNA divided by total counts of all hairpins at day 14) ÷ (Hairpin counts of given shRNA at day 0 divided by total counts of all hairpins at day 0).

### Generation of COMMD3-shRNA lines

Constitutive shCOMMD3 expressing human T-47D and syngeneic 4T07 cell lines were established using human (pGIPz shRNAmir30 vector, Open Biosystems) and mouse (PLKO.1 vector; Sigma), COMMD3-specific shRNAs respectively. A standard lentiviral protocol using PEI method as described above was used to generate the respective cell lines. shRNA sequences used in this study are shown in Table S3.

### Colony formation assays and cell cycle analysis

Cells were seeded in regular tissue culture plates and incubated for 14 days to determine clonogenic potential. The colonies were fixed with 0.05% crystal violet for 30 min, washed and dried. Representative images are shown in figures. Cell cycle analysis was determined by using flow cytometry analysis of cells stained with propidium iodide and analyzed using ModFit LT 4.0 software as described previously [32].

#### Immunoblotting assay

Immunoblotting was performed as described previously [33] in which cells were lysed in Urea Buffer [8 M urea, 1% SDS, 100 mM NaCl, 10 mM Tris (pH 7.5)]. Immunodetection was performed using indicated antibodies listed above in conjunction with a horseradish peroxidase-conjugated anti-rabbit or mouse secondary antibodies (Amersham, GE Healthcare).

#### Immunofluorescence

Cells were seeded on 0.1% poly-l-lysine-coated coverslips and fixed for 15 min in 4% formaldehyde in PBS, permeabilised in 0.05% Triton X-100-PBS for 15 min and blocked in 2% filtered bovine serum albumin (BSA). Primary antibodies were diluted in blocking solution and incubated with slides overnight at 4 °C. Alexafluor conjugated secondary antibodies were diluted 1/300 in blocking solution and stained for 45 min at 37 °C in humidifier chamber. Slides were washed, counterstained with DAPI (diluted 1/500 in blocking buffer, stock 1 mg/ml) and mounted in Prolong Gold. Slides were analyzed using GE DeltaVision Deconvolution microscope. Images were analyzed using Image J.

#### RNA sequencing analysis

Sequencing was carried out at the Institute for Molecular Bioscience Sequencing Facility, The University of Queensland. RNA libraries were made using the Illumina TruSeq Stranded Total RNA (Ribo-Zero GOLD) library preparation kit. Paired-end sequencing was performed using the NextSeq 150 cycle High Output run (2 × 75 bp). A minimum of 25 × 10^6^ reads were obtained for each sample. Sequence reads were trimmed for adapter sequences using Cutadapt and aligned to the mm10 assembly using STAR aligner. The read counts per gene were estimated using RSEM and were utilised to determine differential gene expression between groups using Bioconductor package 'edgeR'. The default TMM normalization method of edgeR was used to normalise read counts between samples. Differentially expressed genes were considered significant if the Benjamini–Hochberg corrected *p*-value was less than 0.01 and a log2 fold change of > 2 [FC > 2].

### Reverse transcriptase–quantitative PCR

RNA was extracted using RNEasy plus Mini Kit (Qiagen, Venlo, Limburg, Netherlands) and cDNA synthesised using the iScript™ cDNA Synthesis Kit (Bio-Rad) according to manufacturer’s instructions. RT-qPCR was performed on a CFX384 Touch™ Real-Time PCR Detection System (Bio-Rad, California, USA) using SYBR Green (Roche) and normalised against β-actin and HPRT1 as internal controls, as described previously [30].

### Measurement of metal ion content

Frozen cell pellets were lysed in unbuffered 0.5% SDS and cleared by ultracentrifugation. Protein concentration in the lysates was measured with BCA and 500ug of each sample was diluted to a final volume of 400uL with additional 0.5% SDS and freeze dried. Aliquots of 400uL 0.5% SDS as blank controls were included as background controls. The samples were assayed by inductively coupled plasma mass spectrometry (ICPMS; Agilent 7700) under routine multi-element operating conditions using a Helium Reaction Gas Cell, according to a previously published method [34]. Briefly, samples were treated with concentrated Nitric Acid (65%, Suprapur, Merck) and digested for six hours at room temperature, then heated at 90°C for 20 min to complete the digestion. The instrument was calibrated using 0, 5, 10, 50, 100 and 500 ppb of certified multi- element ICPMS standard calibration solutions (ICP-MS-CAL2-1, ICP-MS-CAL-3 and ICP-MS-CAL-4, Accustandard) for a range of elements, and a certified internal standard solution containing 200 ppb of Yttrium (Y89) as an internal control (ICP-MS-IS-MIX1-1, Accustandard). Cellular copper levels are presented as a ratio of cellular magnesium content.

### In vivo* tumour growth*

All experiments were in accordance with the guidelines of, and approved by the QIMR Berghofer Medical Research Institute Animal Ethics Committee, as described previously [33]. Briefly, 5–6 week old female Balb/C mice were housed in standard conditions with a 12 h light/dark cycle and free access to food and water. For mammary fat pad injections, 100,000 murine 4T07 cells were prepared in PBS and injected into the inguinal mammary fat pad of 6 week old Balb/C mice. Tumour growth was measured thrice weekly by caliper measurements. To calculate tumour area the following formula was used: tumour area = B*S where B = largest tumour measurement and S = the smallest, based on two-dimensional caliper measurements.

### Statistical analysis

All comparisons between samples were evaluated using the two-tailed non-parametric Mann–Whitney test, one-way or two-way ANOVA with Bonferroni post hoc testing unless otherwise stated in figure legends using GraphPad Prism v8.0 (GraphPad Software, LaJolla, CA, USA). Where applicable, statistical significance is denoted by * for *P* ≤ 0.05; **: *P* ≤ 0.01; ***: *P* ≤ 0.001. Data are expressed as mean ± standard error (SEM) or standard deviation (SD).

## Results

### shRNA screens identified known and novel regulators of breast cancer growth

**Fig. 1:**
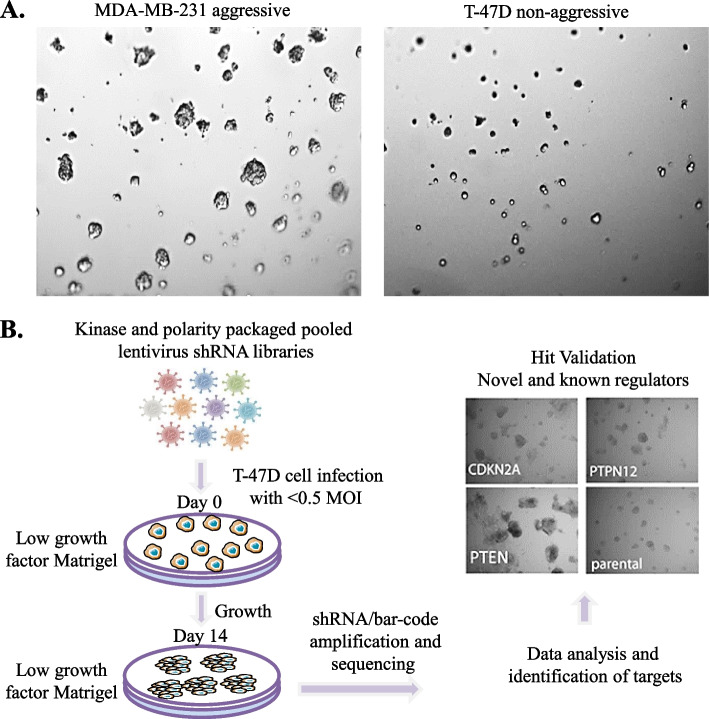
Pooled shRNA screen identifies several genes involved in breast cancer tumourigenesis. **A**. Establishment of the 3D on-top assay in 2% serum with growth factor reduced Matrigel. This method facilitates preferential acini outgrowth of MDA-MB-231, but not T-47D breast cancer cells. **B**. Schematic of workflow showing shRNA screening in T-47D cells. T-47D cells were transduced at an MOI of < 0.5, selected in puromycin for 3 days. For the Polarity and Kinome libraries, 1.5 × 10^6^ selected T-47D cells were plated on a T75 flask of Growth Factor Reduced (GFR) matrigel overlaid with DMEM 2% FCS. Day zero samples were frozen and Day 14 acini were collected by dissolving the matrigel in ice cold versene and pelleting the cells. Genomic DNA was prepared from day 0 and day 14 samples for amplification, sequencing and quantification of shRNA sequences. The abundance of hairpins before and after acini culture was compared to detect enrichment

We set out to identify genes that facilitate spheroid growth from a low proliferative and non-invasive state into larger and more aggressively spreading clusters that models when dormant tumour cells resume growth. We adopted the 3D culture model described by Barkan et al [21], in which cancer cells of interest are cultured at low density on top of a laminin and collagen rich ECM (Matrigel) that mimics the tumour microenvironment more closely than standard 2D cell culture. We tested different serum concentrations to identify a concentration that preferentially supported acini outgrowth of invasive basal MDA-MB-231 cells, compared to non-invasive luminal T-47D cells, settling on 2% foetal calf serum (FCS) and growth factor reduced (GFR) Matrigel (data not shown). Use of low serum concentration and GFR Matrigel allows discrimination between cells with strong metastatic capacity compared to those that display a dormant phenotype *in vivo* [21]. When cultured in 2% FCS, MDA-MB-231 cells form large colonies with projections, indicative of an invasive phenotype, while T-47D cells remain as small rounded colonies (Fig. 1A). We then asked if shRNA-mediated depletion of known tumour suppressors would facilitate aggressive acini formation in T-47D cells. During assay development, we showed that cells depleted for *PTEN*, *PPP2R1B*, and *CDKN2A* formed larger acini (Fig. S1), indicating that this approach was able to enrich for cells with enhanced growth potential of T-47D cells in these nutrient poor conditions.

**Fig. 2:**
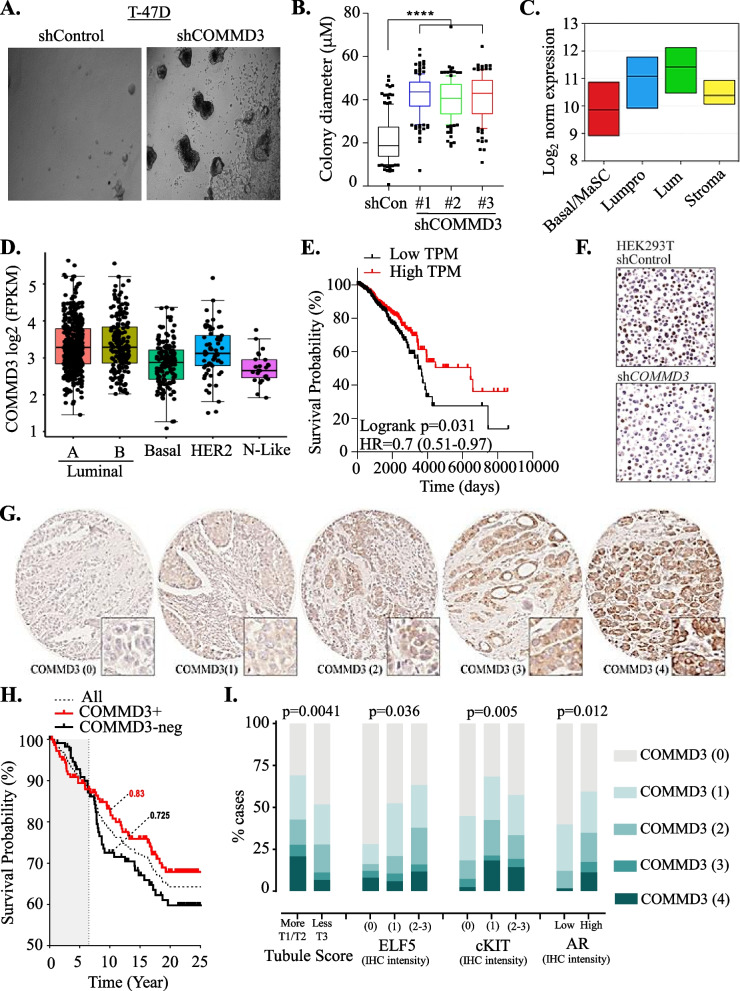
COMMD3 low expression correlates with poor overall survival in breast cancer patients. **A**. T-47D cells were transduced with pGIPz shControl or representative shCOMMD3 hairpin (V2LHS_28871) and selected for 48 h. Cells were plated on GFR matrigel in 2% FCS and grown for 14 days prior to imaging. **B**. Box plot depicting acini size upon COMMD3 depletion measured on day 14; *n* = 2, more than 100 acini were counted. **C**. Analysis of *COMMD3* mRNA levels in FACS-sorted subtypes of human mammary epithelium, taken from Lim et al. (36). Basal/MaSC – basal and stem cells; Lumpro – luminal progenitor cells; Lum – mature luminal cells; Stroma – stromal cells (including fibroblasts). **D**. Box plot depicting *COMMD3* mRNA expression in TCGA breast cancer samples, grouped according to PAM50 subtype (http://tumoursurvival.org). N-Like is normal-like. **E**. Kaplan–Meier survival analysis of the relationship between *COMMD3* mRNA expression and breast cancer patients' clinical outcome using the TCGA RNAseq dataset. *COMMD3* expression stratified overall survival. **F**. Representative images of COMMD3 antibody optimisation by immunohistochemistry (IHC) in HEK293T cells after transfection with the shRNA COMMD3 or control vector. **G**. Representative images of COMMD3 expression in breast cancer tissues on the TMA. Examples of 0–4 + TMA core staining are shown. **H**. Kaplan–Meier survival analysis of the relationship between COMMD3 protein levels and outcome for ER + HER2-negative tumours (luminal A with low Ki67 staining) on the TMA. COMMD3 expression stratified overall survival. **I**. Quantification of COMMD3 protein association with tubule formation, ELF5, c-Kit and AR staining that were assessed for this human breast cancer TMA previously [25–29].

We then completed two screens using pooled shRNA libraries, one encoding kinases and the other curated to include targets and family members of genes implicated in cell polarity (created by a scientific community associated with the Victorian Centre for Functional Genomics, Peter MacCallum Cancer Centre, Melbourne). Libraries encoding either kinases or cell polarity genes were chosen because individual protein from both libraries have been proposed to function as tumour suppressors or oncogenic promoters as well as being implicated in cell migration and invasion processes. T-47D cells were transduced at < 0.5 MOI to reduce the chance of multiple integration of shRNA vectors per cell and puromycin selected for 3 days, followed by culturing on GFR Matrigel for 14 days. To identify changes in shRNA abundance, genomic DNA isolated from day zero (starting material) and day 14 acini was used to amplify shRNA sequences as per standard methods [31, 35]. Relative hairpin read counts were used to quantify enrichment of shRNAs from day zero to day 14 of 3D culture, and when normalised across the screen (Table S1, see method section), allowed us to calculate the relative enrichment of specific shRNAs on day 14 compared to day zero (Fig. 1B). Genes with two or more hairpins that were > twofold enriched on day 14 acini culture compared to day zero were identified (Table 1, Table S1), and the selection of genes (i.e. COMMD3, Evi2A, ACVR1B, EphA10, AXL, KHK, ITK, ARHGAP28, DLG2) for further validation was based on literature searches for their association with cancer progression and selected genes were tested in a single hairpin per well approach. Specific depletion of COMMD3 confirmed its potential role as a tumour suppressor (Fig. 2A), while other genes, including several known regulators (AXL, ACVR1B, and DLG2) were also potential suppressors of the invasive phenotype in T-47D cells, showing enhanced growth defined by colony size when the gene was knocked down (Fig. S2, Table 1 and Table S1).

**Table 1:** Genes whose loss facilitates aggressive acini growth in breast cancer

Genes (Kinome and Polarity library)	Number of hairpins show > 2 fold ratio enrichment
DCLK1, DLG2	4
CLK2, **COMMD3**, EXOSC10, FRK, HUNK, MAPK9, PIP4K2B, PRPF4B, PSKH2, SGK3, TSSK1B, WNK3	3
ACR2A, ARHGEF9, BRAF, CAMKk2, CDC42BPB, CHEK1, CSNK1A1/3,DYRK1B, FPGT, HIPK4, HK2, IKBKE, IRAK1, ITGA6, LIMK3, MAP3K2/3/9, MAP4K4/5, MAPK15, MAPK3/9, MAST2, MERTK, MMP3, MYLK2, MYO3A, NBL1, NEK3/8/9, NUCKS1. PAK6, PBK, PDK1, PHKG1, PI4K2A, PIK3C2A, PIK3R1, PIP4K2B, PKN1, PRKAR2A, PRKD1, PRPF4B, PRPS2, PSKH2, RAF1, RIOK3, RIPK3, RNASEL, SGK3, SMG1, SRC, SRMS, STK11, STK40, TAB1, THP2, TLK2, TNIK, TP53RK, ULK4, WNK2, WNT16	2

### COMMD3 as novel tumour suppressor in breast cancer

To gain further insight into candidate gene regulation from our screen, we focused our attention on the poorly characterised gene *COMMD3*. COMMD3 belongs to family of ten proteins (COMMD1-10), characterised by a structurally conserved, C-terminal COMM protein interaction domain, but possessing divergent N-termini [36]. The canonical family member COMMD1 is a tumour suppressor with cytoplasmic roles controlling vesicular trafficking, and nuclear roles restraining HIF-1α and NFkB activity [37, 38]. We found that shRNA-mediated depletion of COMMD3 using 3 hairpins resulted in cells with larger acini than the shControl cells (Fig. 2B). This observation is consistent with our hypothesis that COMMD3 loss is responsible for more aggressive and invasive acini outgrowth in breast cancer. We next investigated COMMD3 transcript levels in normal human breast and breast tumours by analysing published gene expression datasets. Utilising human transcriptomic data published by Lim et al. (36), different mammary epithelial subtypes were assessed for *COMMD3*, with expression being highest in mature luminal epithelial cells (Lum) and lowest in the basal/mammary stem cells (basal/MaSC) (Fig. 2C). A consultation with the human protein atlas (which is comprised of three independent normal breast tissue samples stained with a different antibody) confirmed strong expression of COMMD3 protein in terminal ductal alveolar units, polarised toward the lumen [39]. In the TCGA breast cancer cohort, *COMMD3* RNA was highest in luminal and HER2 + tumours, compared to basal- and normal-like subtypes (Fig. 2D). Lower tumour expression of *COMMD3* mRNA was associated with a relatively lower probability of survival in the TCGA cohort (Fig. 2E). Analysis of the KMPlotter database showed that these trends were upheld in a range of systemic therapy subgroups (Fig. S3).

To confirm that expression of COMMD3 protein in the tumour cell compartment is associated with disease-specific survival, we performed immunohistochemistry (IHC) analysis of an independent cohort using a commercially available antibody that we first validated on formalin-fixed, paraffin-embedded HEK293T cell pellets (Fig. 2F), showing visibly reduced staining in sh*COMMD3* compared to control cultures. A modified COMMD3 IHC protocol was then applied to a consecutive series of invasive breast tumours sampled in tissue microarrays (Table 2). This revealed a range of protein levels from negative through to strongly positive, and heterogeneity within individual samples. Staining was quantitated using an IHC score (intensity x stained area), then distilled into five categories representing increasing levels of overall expression in the tumour compartment (Fig. 2G). Consistent with the transcript analyses, COMMD3 protein levels were significantly higher in ER + and HER2 + tumours compared to TNBC (Table 3). Interestingly, COMMD3 was inversely associated with the tubule score component of histological grade; that is, tumours that retain some of the polarization and alveolar-like structures typical of normal breast tissue were more likely to express higher levels of COMMD3 (Table 3; ChiSq *p* = 3.0E^−04^).

**Table 2:** Baseline characteristics of COMMD3 IHC cohort

***Mean follow-up (dead)***		6.18 yr	Feature		n
***Mean follow-up (alive)***		17.4 yr	*Receptor subtypes*	ER + /HER2 +	27
***Mean age at dx***		58.4 yr		ER + /HER2–	320
**Feature**		n		ER–/HER2 +	29
***Grade***	1	61		ER–/HER2–	77
	2	232	*COMMD3 IHC score*	0	200
	3	164		-1	111
***Stage***	I	28		-2	81
	II	104		-3	27
	III	14		-4	44

*Abbreviations*: *dx* diagnosis, *IHC* immunohistochemistry, *yr* years

**Table 3:** Association of COMMD3 expression with histopathological parameters in breast cancer

Parameter	q Category	no. cases						% cases					*p* value
	COMMD3 IHC score u	0	1	2	3	4	total	0	1	2	3	4	
Age at diagnosis	< 50 years	139	74	56	11	27	307	45.30%	24.10%	18.20%	3.60%	8.80%	*ns*
	≥ 50 years	54	30	21	12	15	132	40.90%	22.70%	15.90%	9.10%	11.40%	
	*total*	*193*	*104*	*77*	*23*	*42*	*439*						
Stage	I	11	8	6	1	2	28	39.30%	28.60%	21.40%	3.60%	7.10%	*ns*
	II	56	22	17	2	7	104	53.80%	21.20%	16.30%	1.90%	6.70%	
	III	5	2	2	1	4	14	35.70%	14.30%	14.30%	7.10%	28.60%	
	*total*	*72*	*32*	*25*	*4*	*13*	*146*						
Grade	1	21	15	8	5	12	61	34.40%	24.60%	13.10%	8.20%	19.70%	5.80E-02
	2	98	52	45	13	24	232	42.20%	22.40%	19.40%	5.60%	10.30%	
	3	80	41	27	8	8	164	48.80%	25.00%	16.50%	4.90%	4.90%	
	*total*	*199*	*108*	*80*	*26*	*44*	*457*						
Mitotic score	1	108	57	48	17	29	259	41.70%	22.00%	18.50%	6.60%	11.20%	5.80E-02
	2	25	15	12	2	12	66	37.90%	22.70%	18.20%	3.00%	18.20%	
	3	65	36	20	7	3	131	49.60%	27.50%	15.30%	5.30%	2.30%	
	*total*	*198*	*108*	*80*	*26*	*44*	*456*						
Tubule score	T1/2	35	28	21	7	22	113	31.00%	24.80%	18.60%	6.20%	19.50%	**3.00E-04**
	T3	164	80	59	19	22	344	47.70%	23.30%	17.20%	5.50%	6.40%	
	*total*	*199*	*108*	*80*	*26*	*44*	*457*						
ER/HER2 subtype	ER-/HER2-	47	18	10	2	0	77	61.00%	23.40%	13.00%	2.60%	0.00%	**5.40E-05**
	ER-/HER2 +	9	3	10	2	5	29	31.00%	10.30%	34.50%	6.90%	17.20%	
	*total*	*56*	*21*	*20*	*4*	*5*	*106*						
	ER + /HER2-	133	80	52	19	36	320	41.60%	25.00%	16.30%	5.90%	11.30%	*ns*
	ER + /HER2 +	8	6	7	3	3	27	29.60%	22.20%	25.90%	11.10%	11.10%	
	*total*	*141*	*86*	*59*	*22*	*39*	*347*						
Histological type	IC (NST)	112	59	62	16	24	273	41.00%	21.60%	22.70%	5.90%	8.80%	5.50E-02
	ILC & variants	29	11	8	5	8	61	47.50%	18.00%	13.10%	8.20%	13.10%	
	mixed ducto-lob	18	13	3	0	4	38	47.40%	34.20%	7.90%	0.00%	10.50%	
	mixed IC + special type	20	9	3	1	2	35	57.10%	25.70%	8.60%	2.90%	5.70%	
	metaplastic	8	3	3	2	0	16	50.00%	18.80%	18.80%	12.50%	0.00%	
	special types	12	12	1	2	6	33	36.40%	36.40%	3.00%	6.10%	18.20%	
	*total*	*199*	*107*	*80*	*26*	*44*	*456*						
Prognostic subgroups	HER2 +	17	9	17	5	8	56	30.40%	16.10%	30.40%	8.90%	14.30%	**4.40E-02**
	ER + (Ki67-low)	114	67	43	14	30	268	42.50%	25.00%	16.00%	5.20%	11.20%	
	ER + (Ki67-high)	11	7	5	2	4	29	37.90%	24.10%	17.20%	6.90%	13.80%	
	TN (basal-like)	35	15	7	2	0	59	59.30%	25.40%	11.90%	3.40%	0.00%	
	TN (non-basal)	8	3	3	0	0	14	57.10%	21.40%	21.40%	0.00%	0.00%	
	*total*	*185*	*101*	*75*	*23*	*42*	*426*						

There were no significant associations with survival in either TNBC or HER2 + groups (not shown), but COMMD3 protein loss in ER + HER2-negative tumours was associated with poorer survival outcomes, particularly luminal A-like cases (defined here by Ki67 staining in ≤ 20% of tumour cells; Fig. 2H). Notably, COMMD3 only stratified survival in this group after about seven years. In other words, for patients with luminal-A-like breast cancer who were still alive seven years after diagnosis, survival probability was lower for tumours lacking COMMD3 (e.g., 72.5% of patients alive at 10 years post-diagnosis, verus 83% for COMMD3 + cases (Fig-2H)). Interestingly, COMMD3 was significantly associated with markers of luminal differentiation and/or fate commitment in the luminal A-like tumours: tubule formation, androgen receptor (AR) [40], ELF5 [41] and cKIT [42] (Fig-2I).

### Loss of COMMD3 enhances tumourigenic potential in breast cancer

**Fig. 3:**
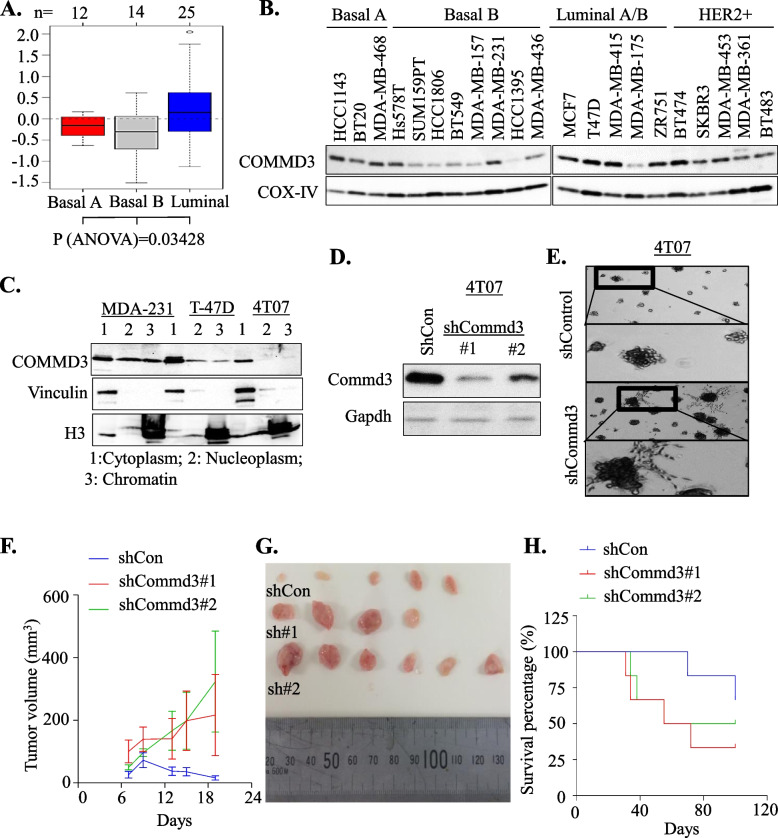
COMMD3 loss causes aggressive tumour formation in breast cancer. **A**. Box plot of COMMD3 transcripts across cell lines grouped in the basal A (red), basal B (grey) and luminal (blue) subgroups based on annotation data from Neve et al. [43]. A *p* value shows difference between basal and luminal determined by ANOVA test. These data were derived from Gene expression-based Outcome for Breast cancer Online (GOBA) analysis tool [44]. **B**. Western blot analysis of COMMD3 protein levels in a panel of human breast cancer lines representing the different subtypes of breast cancer. COX-IV used a loading control. **C**. Western blot analysis showing COMMD3 protein levels in different subcellular fractions of breast cancer cell lines. Vinculin and H3 were used as markers of the different fractions. Cytoplasmic (1), and nucleoplasmic (2) and chromatin (3). **D**. 4T07 cells were transduced with pGIPz shControl or two different shCOMMD3 hairpin sequences and selected for stable expression. The efficiency of COMMD3 depletion was assessed by western blotting. **E**. 4T07 cells expressing the shcontrol or shCommd3 #1 were plated on GFR matrigel in 2% FCS and grown for 14 days prior to imaging. **F**. Tumour growth curves after implantation of 4T07 cells into the inguinal mammary gland of female Balb/C mice. Bars show mean ± SD. *N* = 6 mice in each group, ANOVA with Holm-Sidak test, *P* value at day 19: shCon vs. shCommd3#1: 0.0444; shCon vs. shCommd3#2: 0.0047. **G**. Representative images of gross morphology of excised tumours at endpoint. **H**. Mouse survival after implantation of 1 × 10^5^ cells into fat pads of female Balb/C mice. *N* = 6 mice in each group. Hazard Ratio (logrank) for shCon vs. shCommd3#1: 0.3070 (95% CI: 0.05854 to 1.611), *p* value: 0.1626; shCon vs. shCommd3#2: 0.4682 (95% CI: 0.07892 to 2.778), *p* value: 0.3912. Median survival of each group: shcon: undefined; shCommd3#1: 63.5 days; shCommd3#2: 77.5 days

Given the enrichment of shCOMMD3 cells in acini from our genetic screen and the robust expression of COMMD3 in well-differentiated, lower grade breast cancers, we next explored the tumour suppressor function of COMMD3 in more detail. We found that the aggressive and invasive basal-like breast cell lines, particularly the basal B lines exhibited lower COMMD3 expression at the transcript level (data from Neve et al. [43]) and at the protein level compared to luminal cell lines (Fig. 3A-B, Fig. S4A). Moreover, subcellular fractionation of COMMD3 in T-47D, MDA-MB-231 and 4T07 breast cancer cell lines demonstrated that COMMD3 is higher in the cytoplasmic compared to nuclear fractions (Fig. 3C). We also observed a low level of COMMD3 in the nuclear chromatin bound fractions, indicating that COMMD3 expression is pan-cellular (Fig. 3C).

To study COMMD3 function in breast cancer, we utilised a non-metastatic 4T07-TGL (tagged with TK-GFP-luciferase) cell line that is syngeneic to Balb/c mice. We generated a panel of shCommd3 cell lines and identified one with strong depletion (hairpin 191,395#1) and one with partial depletion (hairpin 201,193#2) of Commd3 (Fig. 3D, Fig. S4B). We found that depletion of mCommd3 did not alter proliferation rate, cell cycle distribution, or colony forming capacity of cells in 2D culture (Fig. S4C-E). However, it did increase acini growth in 3D culture (Fig. 3E). To explore the role of mCommd3 tumour growth in vivo, a low number of tumour cells (100,000 cells) was engrafted into the mammary fat-pad of Balb/C mice. Interestingly the cell lines with nearly complete or partial depletion of Commd3 had similar growth characteristics (Fig. 3F and G), indicating Commd3 haplo-insufficiency. Moreover, the shCommd3 cells formed palpable tumours with a shorter latency period and larger tumours at endpoint than their respective shControl tumours (Fig. 3H and Fig. S4F). Collectively, our data provided strong evidence that COMMD3 expression suppresses breast cancer growth in 3D culture and in vivo.

### Whole-transcriptome profiling identifies COMMD3-regulated networks in breast cancer

**Fig. 4:**
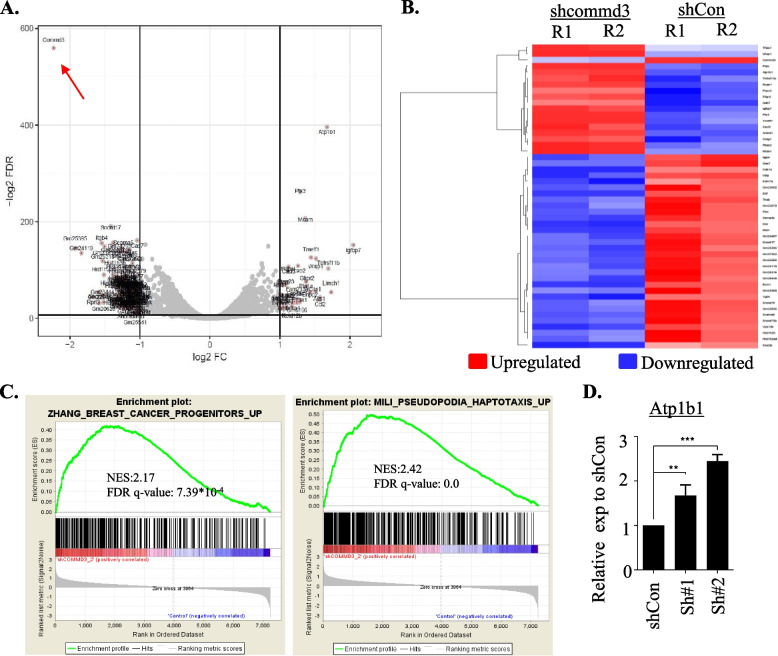
Transcriptional profiling identified Commd3 regulated pathways in breast cancer. **A**. Volcano plot of shCOMMD3 vs. shcontrol differential expression analysis. Displayed is the log2 FC (x-axis) in dependency to the negative log2 FDR adjusted *p*-value. Horizontal black line indicates a log2 transformed FDR of 0.01 and vertical lines a log2 fold change of -2 and + 2. **B**. Heatmap of up and downregulated genes in 4T07-TGL (tagged with TK-GFP-luciferase) shCommd3 relative to shControls. One shControl and two independent hairpins were analysed by RNAseq in experimental duplicate. **C**. Gene Set Enrichment Analysis showing breast cancer progenitors and pseudopodia gene signatures are enriched upon COMMD3 depletion in 4T07 cells. **D**. PCR validation of expression levels of Atp1b1 in shCommd3 cells. Validation was completed on samples independent of the RNAseq data. Transcript abundance of Atpb1 was normalized to actin. Bars show mean ± SEM, *n* = 3; shCon vs. shCommd3#1: *p* = 0.0074; shCon vs. shCommd3#2: *p* = 0.0005

COMMD1 has been shown to inhibit NFkB and HIF1α mediated transcription and is also involved in copper homeostasis [45]. There is more limited evidence that COMMD3 might also be involved in similar processes [46–48]. To gain insight into COMMD3 regulated transcriptional networks, we analysed the transcriptome of 4T07 shControl compared to cells expressing one of two independent shCOMMD3 hairpins in experimental duplicate (two independent samples each of one control and two shRNA hairpins, both with > 90% mCommd3 knockdown). As expected, m*Commd3* was the most downregulated gene in our analysis, confirming extensive depletion (Fig. 4A). Compared to control cells, shCommd3 cells displayed 202 genes with > 1.5-fold downregulation and 43 genes with 1.5-fold upregulation (Fig. 4A, B and Table S2). Consistent with our immunohistochemical analysis of the breast cancer TMA and the shRNA depletion phenotype, gene set enrichment analysis (GSEA) found that shCommd3 cells were enriched for breast cancer progenitor and pseudopodia haptotaxis signatures (Fig. 4C). Such transcriptional reprogramming could underpin the outgrowth of breast cancer cells with Commd3 loss. We did not observe changes in NFkB and HIF1α mediated transcription as previously reported for COMMD1, suggesting that COMMD3 is part of neither NFkB nor HIF1α networks. Notably, the most highly upregulated gene (2.4-fold, *p* = 2.74E-06) in shCommd3 cells was the Na + /K + transporter *ATP1B1* (Fig. 4A). Upregulation of *ATP1B1* transcripts was validated independently, revealing 2–threefold increase upon Commd3 loss in 4T07 cells (Fig. 4D). Since ATP1B1 transcript has been reported to be upregulated in response to copper overload [46, 49], this provides an initial hint that, similar to Commd1, Commd3 deficiency causes changes in copper homeostasis in breast cancer.

### Commd3 loss enhances copper overload in breast cancer

**Fig. 5:**
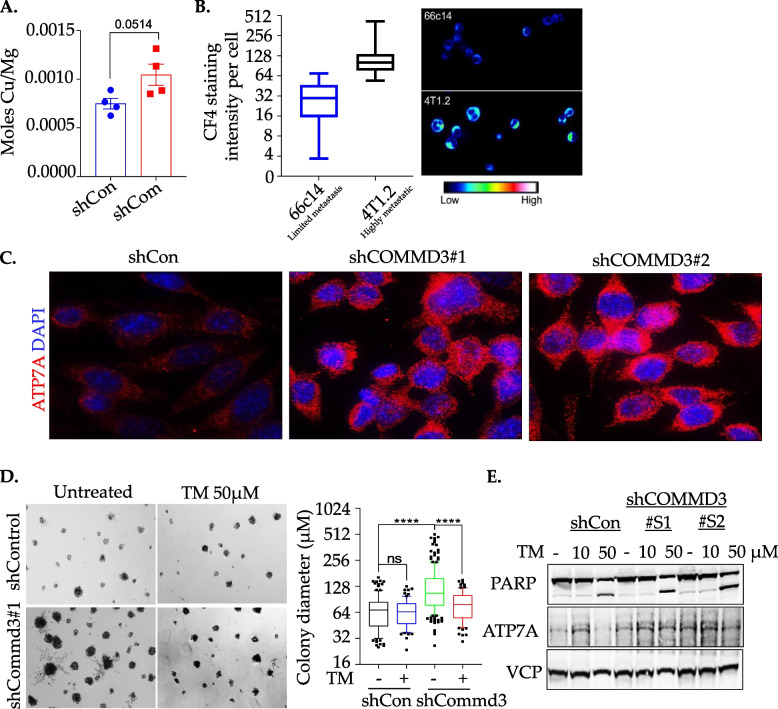
Copper chelation sensitises shCOMMD3 cells. **A**. Copper levels in both shCOMMD3 and shcontrol 4T07-TGL (tagged with TK-GFP-luciferase) cells evaluated using Agilent 7700 ICPMS instrument. The levels were expressed as Moles of Cu/Mg of protein. **B**. CF4 staining to reveal Cu + abundance in 4T1.2 and 66cl4 cells. The intensity per cell was calculated to reflect the level of Cu + abundance. **C**. Protein levels of ATP7A level in both shCOMMD3 and shcontrol 4T07-TGL (tagged with TK-GFP-luciferase) cells. Nuclei are stained with DAPI. **D**. Representative images of 4T07 shControl and shCommd3 acini were grown on GFR matrigel in 2% FCS in the presence or absence of 50 µM tetrathiomolybdate (TM). Acini size was quantified by measuring diameters on day 10; *n* = 2. **E**. Western blot analysis showing the effect of different concentrations of tetrathiomolybdate (TM) on PARP and ATP7A levels at 24 h. Valosin containing protein (VCP) used as a loading control

Given the established role of COMMD1 in mediating intracellular copper levels [45] and the fact that ATP1B1 can be upregulated by copper toxicosis [44, 47], we hypothesised that COMMD3 deficient cells might also have changes in copper metabolism, involving upregulation of *ATP1B1*. We therefore assessed intracellular copper abundance by ICP-MS of whole cell lysates. Although not statistically significant, we found that the shCommd3 4T07 cells have slightly increased intracellular copper levels relative to shControl (Fig. 5A). Copper accumulation was also assessed in two lines of the murine 4T1 metastasis model (66c14 and 4T1.2) using the Cu^+^-specific fluorescence-based sensor, Copper Fluor-4 (CF4). The highly metastatic 4T1.2 line in which Commd3 is lowly expressed had significantly higher levels of Cu^+^ compared to the poorly metastatic 66cl4 cells (Fig. 5B). ATP7A, the major copper-transporting ATPase that regulates copper homeostasis [50] was markedly increased in shCommd3 depleted 4T07 cells compared to shcontrol cells (Fig. 5C), further indicating that Commd3 controls copper homeostasis through ATP7A. To see if depletion of copper would reduce the invasive phenotype of Commd3 deficient cells, we employed the clinically approved copper chelator Tetrathiomolybdate (TM). Treatment of shCommd3 acini with TM significantly reduced the size of acini (Fig. 5D), concomitant with marked induction of apoptosis, as assessed by cleaved PARP (Fig. 5E). However, we did not see any change in ATP7A levels upon TM treatment. These results indicate that copper chelation could be controlling the aggressive and invasive phenotype of COMMD3 deficient breast cancers.

## Discussion

In the current study, we adopted the 3D on-top assay in low serum conditions to identify genes responsible for controlling tumour invasiveness in breast cancer. Through shRNA-mediated depletion screens, we identified several known and novel proteins that negatively regulate tumourigenesis. In particular, here, we demonstrated that COMMD3 loss is linked to aggressive breast tumour growth.

COMMD3 belongs to a family of 10 COMM Domain-containing proteins that are widely conserved and share a unique domain in the carboxy-terminus (COMMD1-10) [48]. However, the expression and function of COMMD3 in human cancer, including breast cancer, is not well understood. We found that depleting COMMD3 in human T-47D cells markedly increased spheroid growth under growth factor reduced conditions. This indicated that COMMD3 is acting as a tumour suppressor in breast cancer. Consistent with these data, we found that COMMD3 is transcriptionally downregulated in aggressive TNBC compared to luminal tumours, and that COMMD3-high patients have prolonged overall survival. Moreover, we found a negative association between high COMMD3 expression and tubule score, ELF5, c-Kit and AR expression in a large cohort of breast cancer cases. Notably COMMD3 is also reported to be lost in B-cell lymphoma [51]. However, contrary to these observations, high levels of COMMD3 confer worse survival in hepatocellular carcinoma [52], indicating tissue-specific roles for COMMD3 in different cancers.

COMMD3 is a poorly characterised protein. Thus, we examined its expression in a large panel of low-passage ATCC breast cancer cell lines. COMMD3 is widely expressed across luminal cell lines and has reduced expression in some TNBC lines of Basal B intrinsic subtype, which is more aggressive. This supports a model where COMMD3 loss is associated with increased aggressive phenotypes. Although COMMD3 depletion did not affect proliferation or cell cycle distribution of 4T07 tumours under standard 2D growth conditions, COMMD3 loss resulted in faster growing tumours, indicating that COMMD3 restrains breast cancer growth in vivo.

COMMD1 has been shown to inhibit NFkB and HIF1α mediated transcription [47, 48]. To further understand the role of COMMD3 in transcription regulation in our model system, we completed RNA sequencing and found that the top gene altered upon COMMD3 loss was *ATP1B1,* which is implicated in regulation of copper homeostasis [46, 49]. An ealier report found that the founding member of the COMMD family, COMMD1, is mutated in canine copper toxicosis [53, 54], hence implicating COMMD proteins in copper homeostasis through association with both ATP7A and ATP7B, key proteins responsible for controlling intracellular Cu(II) accumulation through trafficking into and out of the cell [55]. Consistent with this, we observed an overload of copper in COMMD3 depleted cells along with an increase in copper levels in the highly metastatic 4T1.2 breast cancer cell line. Given the role of ATP7A in transporting Cu(II) into cells, we also determined its level and found increased levels of ATP7A protein in COMMD3 depleted cells, thus indicating that COMMD3, like COMMD1, also controls ATP7A levels.

Increased copper is implicated in cancer development through regulation of angiogenesis, remodelling the tumour microenvironment and contributing to chemotherapy resistance [56, 57]. Copper overload has been linked to disease progression and metastasis, particularly in TNBC, where copper depletion significantly inhibits tumour growth and provides survival benefits to patients [34, 58, 59]. Cells with Commd3 loss contained increased copper and ATP7A, and treatment with a copper chelator caused a significant reduction in spheroid growth, along with induction of apoptosis. This indicates that copper has a major role in breast cancer tumourigenesis and that COMMD3 acts to maintain copper homeostasis.

## Conclusion

For the first time, we report a novel role of COMMD3 as a negative regulator of tumour growth in breast cancer. We provide evidence that COMMD3 controls copper levels in cancer cells. Elevated copper is known to drive solid tumour growth [57]. Thus, metal homeostasis is directly linked to cancer aggressiveness [57]. Previous research has explored transcriptional roles for COMMD proteins in non-cancer cells, but only examined a very small subset of transcripts. This does not adequately assess the true complexity of the situation. We showed that COMMD3 is one of the many genes that can control tumour aggressiveness and provide evidence that copper chelation in COMMD3 low expressing cells would be a potential strategy to counteract disease progression and metastasis of breast cancer.

## Supplementary Information

**Additional file 1. Figures S1** and **S2**: T-47D cells were transduced with pGIPz shRNAs corresponding to the indicated tumour suppressor or candidate gene. Cells were plated on top of GFR matrigel in 2% FCS DMEM and cultured for 14 days with medium changes twice weekly. Each well shows a representative image of a individual hairpin. **Figure S3**: Kaplan-Meier survival analysis of the relationship between COMMD3 mRNA expression and clinical outcome in breast cancer patients treated with or without chemotherapy using the KMplotter dataset (http://kmplot.com/). COMMD3 expression was stratified against overall survival, relapse-free survival and and distant metastasis-free survival. **Figure S4**: **A**. COMMD3 mRNA expression profile in a panel of breast cancer cell lines determined using Neve et al. (41), dataset through GOBO website (http://co.bmc.lu.se/gobo/). Basal A (red), basal B (grey) and luminal (blue) subgroups. **B**. COMMD3 depletion efficiency using different shRNA hairpins in 4T07 cells. **C**. Percentage of each phase of cell cycle upon COMMD3 depletion in 4T07 cells. Graph represents the mean ± SD of two independent experiments. **D**. 4T07 cells were stained with CellTrace™ CFSE for 3 days and loss of CFSE fluorescence intensity was plotted as a line graph. **E**. Representative images showing colony forming capacity of shcontrol and shCOMMD3 depleted 4T07 cells. **F. **Weight of excised 4T07 tumours at endpoint. Bars show mean +/-SEM. *n*=6 mice per group. T test was used.

**Additional file 2.**

**Additional file 3.**

**Additional file 4.**

## Data Availability

The datasets generated and/or analyzed during the current study are included in this published article (and its supplementary information files) and all the raw data available from the corresponding author on reasonable request.

## References

[1] Sung H (2021). Global cancer statistics 2020: GLOBOCAN estimates of incidence and mortality worldwide for 36 cancers in 185 countries. CA Cancer J Clin.

[2] Torre LA, Islami F, Siegel RL, Ward EM, Jemal A (2017). Global Cancer in Women: Burden and Trends. Cancer Epidemiol Biomarkers Prev.

[3] an overview of the randomised trials. Early Breast Cancer Trialists’ Collaborative, G. Effects of chemotherapy and hormonal therapy for early breast cancer on recurrence and 15-year survival. Lancet. 2005;365:1687–717. 10.1016/S0140-6736(05)66544-0 15894097

[4] Demicheli R, Terenziani M, Bonadonna G (1998). Estimate of tumor growth time for breast cancer local recurrences: rapid growth after wake-up?. Breast Cancer Res Treat.

[5] Cancer Genome Atlas, N (2012). Comprehensive molecular portraits of human breast tumours. Nature.

[6] Perou CM (2000). Molecular portraits of human breast tumours. Nature.

[7] Hoeferlin LA, Chalfant CE, Park MA (2013). Challenges in the treatment of triple negative and her2-overexpressing breast cancer. J Surg Sci.

[8] Kalimutho M (2015). Targeted therapies for triple-negative breast cancer: combating a stubborn disease. Trends Pharmacol Sci.

[9] MacMillan CD, Chambers AF, Tuck AB. Progression of early breast cancer to an invasive phenotype. breast cancer metastasis and drug resistance. Breast Cancer Metastasis Drug Resist. 2013;143–159.

[10] Cowell CF (2013). Progression from ductal carcinoma in situ to invasive breast cancer: revisited. Mol Oncol.

[11] Duijf PHG (2019). Mechanisms of genomic instability in breast cancer. Trends Mol Med.

[12] Kalimutho M (2019). Patterns of genomic instability in breast cancer. Trends Pharmacol Sci.

[13] Dai X, Xiang L, Li T, Bai Z (2016). Cancer hallmarks, biomarkers and breast cancer molecular subtypes. J Cancer.

[14] Colaprico A (2020). Interpreting pathways to discover cancer driver genes with Moonlight. Nat Commun.

[15] Kapalczynska M (2018). 2D and 3D cell cultures - a comparison of different types of cancer cell cultures. Arch Med Sci.

[16] Feng Y (2018). Breast cancer development and progression: Risk factors, cancer stem cells, signaling pathways, genomics, and molecular pathogenesis. Genes Dis.

[17] Marcotte R (2016). Functional genomic landscape of human breast cancer drivers, vulnerabilities, and resistance. Cell.

[18] Yu C (2016). High-throughput identification of genotype-specific cancer vulnerabilities in mixtures of barcoded tumor cell lines. Nat Biotechnol.

[19] Peinado H (2017). Pre-metastatic niches: organ-specific homes for metastases. Nat Rev Cancer.

[20] Weigelt B, Ghajar CM, Bissell MJ (2014). The need for complex 3D culture models to unravel novel pathways and identify accurate biomarkers in breast cancer. Adv Drug Deliv Rev.

[21] Barkan D (2008). Inhibition of metastatic outgrowth from single dormant tumor cells by targeting the cytoskeleton. Cancer Res.

[22] Gyorffy B (2010). An online survival analysis tool to rapidly assess the effect of 22,277 genes on breast cancer prognosis using microarray data of 1,809 patients. Breast Cancer Res Treat.

[23] Cerami E (2012). The cBio cancer genomics portal: an open platform for exploring multidimensional cancer genomics data. Cancer Discov.

[24] Gao J (2013). Integrative analysis of complex cancer genomics and clinical profiles using the cBioPortal. Sci Signal.

[25] Saunus JM (2022). Epigenome erosion and SOX10 drive neural crest phenotypic mimicry in triple-negative breast cancer. NPJ Breast Cancer.

[26] Kalita-de Croft P (2020). Clinicopathologic significance of nuclear HER4 and phospho-YAP(S(127)) in human breast cancers and matching brain metastases. Ther Adv Med Oncol.

[27] Adwal A (2020). Tradeoff between metabolic i-proteasome addiction and immune evasion in triple-negative breast cancer. Life Sci Alliance.

[28] Raghavendra A (2018). Expression of MAGE-A and NY-ESO-1 cancer/testis antigens is enriched in triple-negative invasive breast cancers. Histopathology.

[29] Hernandez-Perez S (2017). DUB3 and USP7 de-ubiquitinating enzymes control replication inhibitor Geminin: molecular characterization and associations with breast cancer. Oncogene.

[30] Anderson AM (2017). The metastasis suppressor RARRES3 as an endogenous inhibitor of the immunoproteasome expression in breast cancer cells. Sci Rep.

[31] Cipponi A (2020). MTOR signaling orchestrates stress-induced mutagenesis, facilitating adaptive evolution in cancer. Science.

[32] Kalimutho M (2011). Satraplatin (JM-216) mediates G2/M cell cycle arrest and potentiates apoptosis via multiple death pathways in colorectal cancer cells thus overcoming platinum chemo-resistance. Cancer Chemother Pharmacol.

[33] Kalimutho M (2018). CEP55 is a determinant of cell fate during perturbed mitosis in breast cancer. EMBO Mol Med.

[34] McAllum EJ (2020). Regional iron distribution and soluble ferroprotein profiles in the healthy human brain. Prog Neurobiol.

[35] Bhatia S (2020). Identifying therapies to combat epithelial mesenchymal plasticity-associated chemoresistance to conventional breast cancer therapies using an shRNA Library Screen. Cancers (Basel).

[36] Healy MD (2018). Structural insights into the architecture and membrane interactions of the conserved COMMD proteins. Elife.

[37] Muller PA (2009). Nuclear-cytosolic transport of COMMD1 regulates NF-kappaB and HIF-1 activity. Traffic.

[38] Laulumaa S, Varjosalo M (2021). Commander Complex-A Multifaceted Operator in Intracellular Signaling and Cargo. Cells.

[39] Uhlen M (2015). Proteomics. Tissue-based map of the human proteome. Science.

[40] Tarulli GA (2019). Androgen receptor signalling promotes a luminal phenotype in mammary epithelial cells. J Mammary Gland Biol Neoplasia.

[41] Lee HJ (2013). Progesterone drives mammary secretory differentiation via RankL-mediated induction of Elf5 in luminal progenitor cells. Development.

[42] Lim E (2009). Aberrant luminal progenitors as the candidate target population for basal tumor development in BRCA1 mutation carriers. Nat Med.

[43] Neve RM (2006). A collection of breast cancer cell lines for the study of functionally distinct cancer subtypes. Cancer Cell.

[44] Ringner M, Fredlund E, Hakkinen J, Borg A, Staaf J (2011). GOBO: gene expression-based outcome for breast cancer online. PLoS One.

[45] Riera-Romo M (2018). COMMD1: a multifunctional regulatory protein. J Cell Biochem.

[46] Chen DS, Chan KM (2009). Changes in the protein expression profiles of the Hepa-T1 cell line when exposed to Cu2+. Aquat Toxicol.

[47] van de Sluis B (2010). COMMD1 disrupts HIF-1alpha/beta dimerization and inhibits human tumor cell invasion. J Clin Invest.

[48] Maine GN, Burstein E (2007). COMMD proteins: COMMing to the scene. Cell Mol Life Sci.

[49] Armendariz AD, Gonzalez M, Loguinov AV, Vulpe CD (2004). Gene expression profiling in chronic copper overload reveals upregulation of Prnp and App. Physiol Genomics.

[50] Kaler SG (2011). ATP7A-related copper transport diseases-emerging concepts and future trends. Nat Rev Neurol.

[51] Ferreira BI (2008). Comparative genome profiling across subtypes of low-grade B-cell lymphoma identifies type-specific and common aberrations that target genes with a role in B-cell neoplasia. Haematologica.

[52] Wang X (2021). Transcriptional analysis of the expression, prognostic value and immune infiltration activities of the COMMD protein family in hepatocellular carcinoma. BMC Cancer.

[53] van De Sluis B, Rothuizen J, Pearson PL, van Oost BA, Wijmenga C (2002). Identification of a new copper metabolism gene by positional cloning in a purebred dog population. Hum Mol Genet.

[54] Klomp AE, van de Sluis B, Klomp LW, Wijmenga C (2003). The ubiquitously expressed MURR1 protein is absent in canine copper toxicosis. J Hepatol.

[55] Materia S, Cater MA, Klomp LW, Mercer JF, La Fontaine S (2012). Clusterin and COMMD1 independently regulate degradation of the mammalian copper ATPases ATP7A and ATP7B. J Biol Chem.

[56] Gupte A, Mumper RJ (2009). Elevated copper and oxidative stress in cancer cells as a target for cancer treatment. Cancer Treat Rev.

[57] Lelievre P, Sancey L, Coll JL, Deniaud A, Busser B (2020). The multifaceted roles of copper in cancer: a trace metal element with dysregulated metabolism, but also a target or a bullet for therapy. Cancers (Basel).

[58] Karginova O (2019). Inhibition of copper transport induces apoptosis in triple-negative breast cancer cells and suppresses tumor angiogenesis. Mol Cancer Ther.

[59] Cui L (2021). Mitochondrial copper depletion suppresses triple-negative breast cancer in mice. Nat Biotechnol.

