# Supplementary material for: COMMD3 loss drives invasive breast cancer growth by modulating copper homeostasis
Source: J Exp Clin Cancer Res. 2023 Apr 18;42:90. doi: 10.1186/s13046-023-02663-8 (PMC10111822; doi:10.1186/s13046-023-02663-8)
Supplement: Supplementary file 1 — Additional file 1. Figures S1 and S2: T-47D cells were transduced with pGIPz shRNAs corresponding to the indicated tumour suppressor or candidate gene. Cells were plated on top of GFR matrigel in 2% FCS DMEM and cultured for 14 days with medium changes twice weekly. Each well shows a representative image of a individual hairpin. Figure S3: Kaplan-Meier survival analysis of the relationship between COMMD3 mRNA expression and clinical outcome in breast cancer patients treated with or without chemotherapy using the KMplotter dataset (http://kmplot.com/). COMMD3 expression was stratified against overall survival, relapse-free survival and and distant metastasis-free survival. Figure S4: A. COMMD3 mRNA expression profile in a panel of breast cancer cell lines determined using Neve et al. (41), dataset through GOBO website (http://co.bmc.lu.se/gobo/). Basal A (red), basal B (grey) and luminal (blue) subgroups. B. COMMD3 depletion efficiency using different shRNA hairpins in 4T07 cells. C. Percentage of each phase of cell cycle upon COMMD3 depletion in 4T07 cells. Graph represents the mean ± SD of two independent experiments. D. 4T07 cells were stained with CellTrace™ CFSE for 3 days and loss of CFSE fluorescence intensity was plotted as a line graph. E. Representative images showing colony forming capacity of shcontrol and shCOMMD3 depleted 4T07 cells. F. Weight of excised 4T07 tumours at endpoint. Bars show mean +/-SEM. n=6 mice per group. T test was used. [file 13046_2023_2663_MOESM1_ESM.pdf]

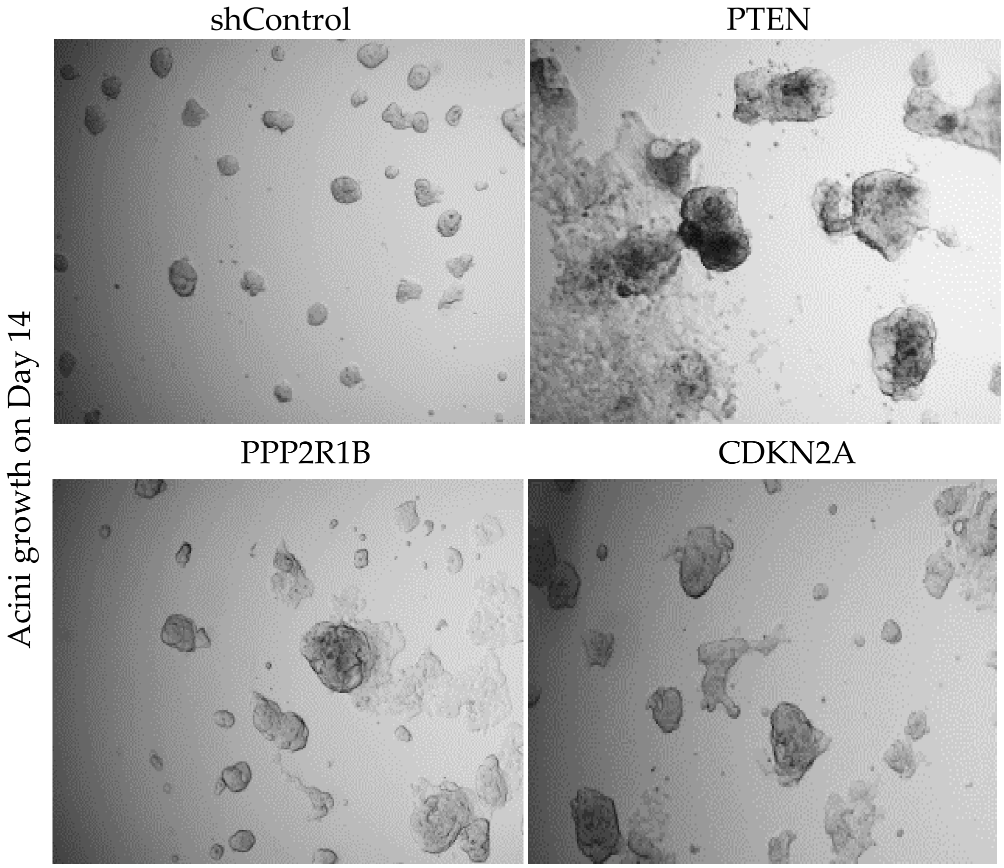

Figure S1

Acini growth on Day 14

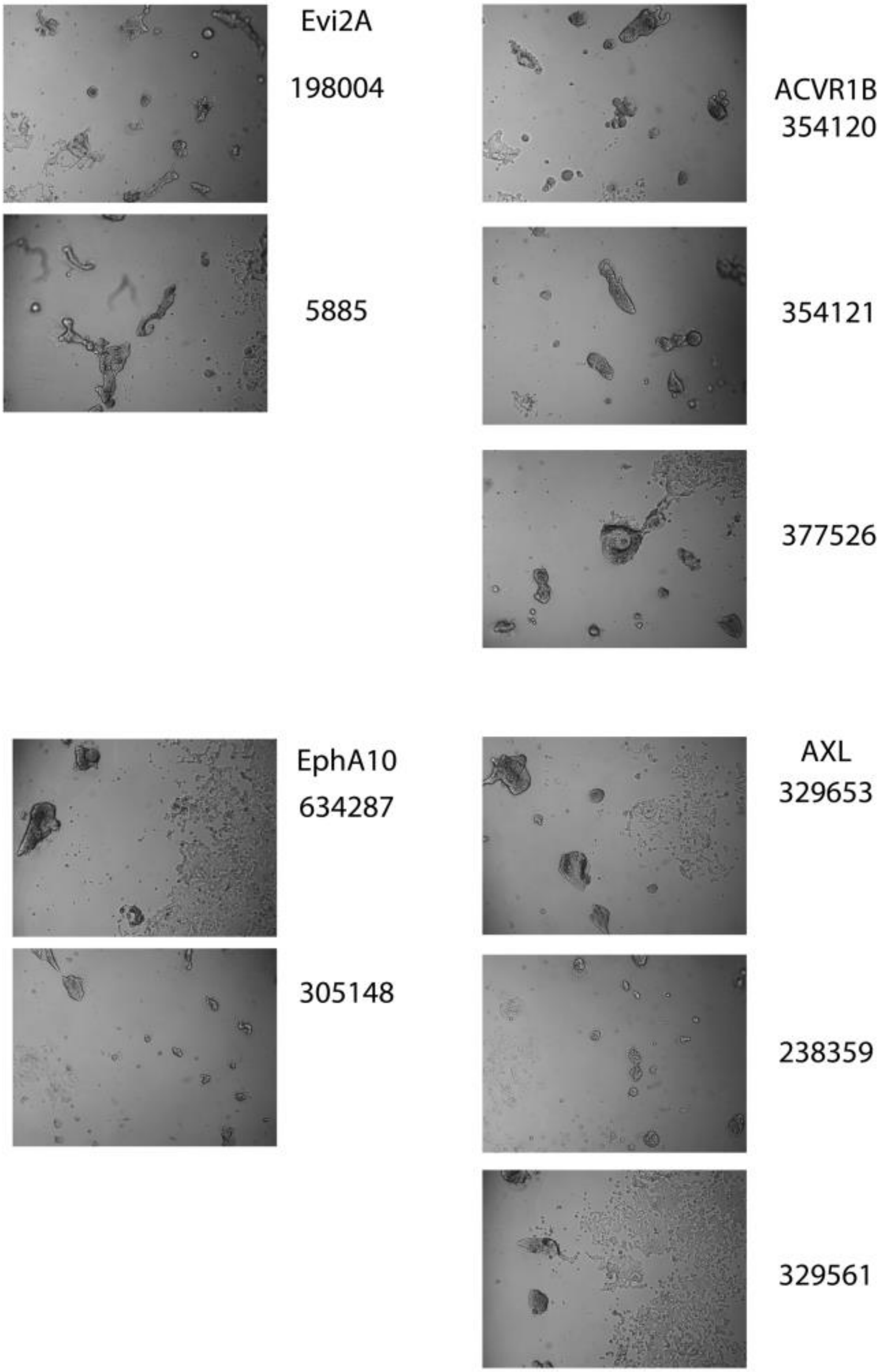

Figure S2

Continued

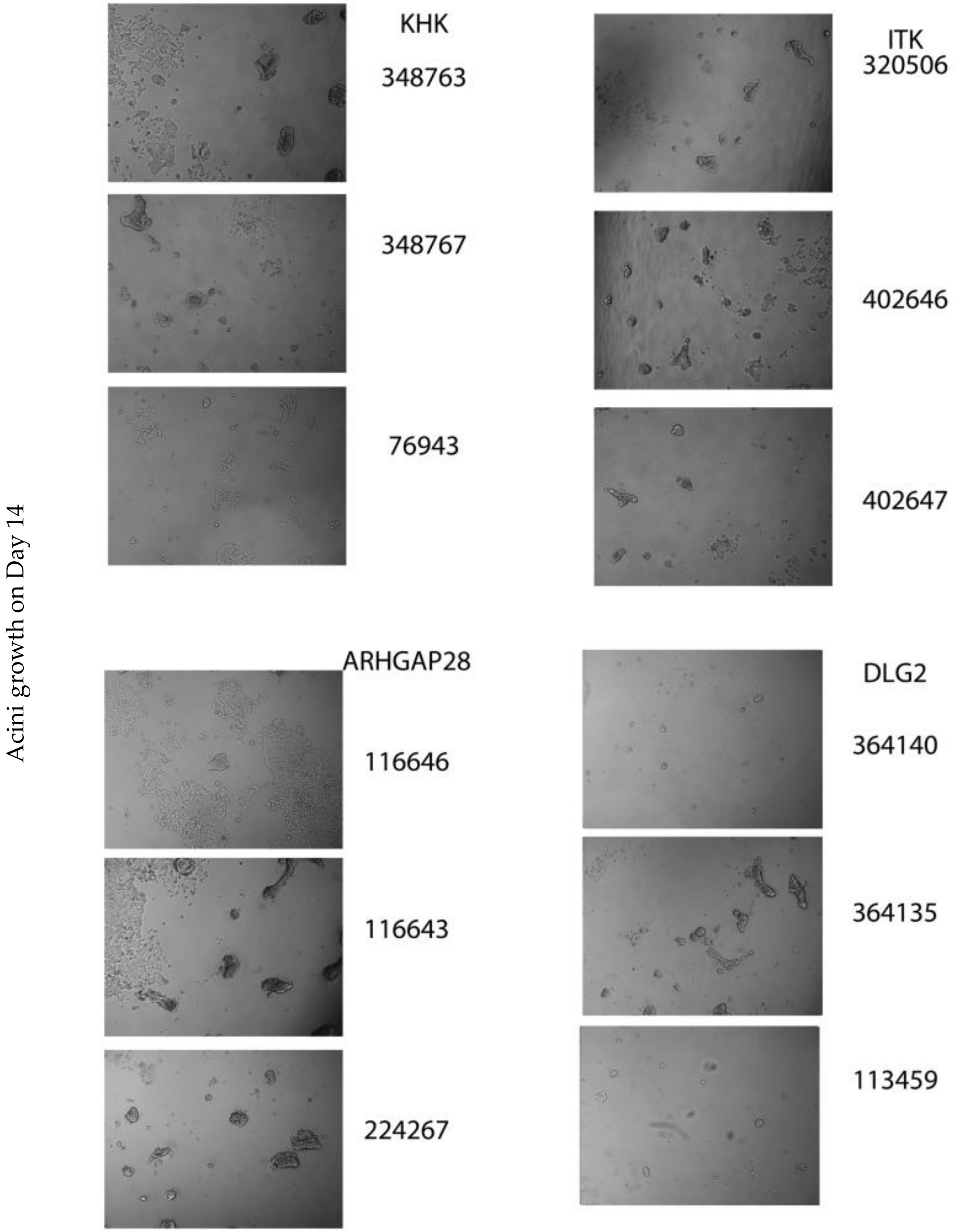
Acini growth on Day 14
KHK
348763
348767
76943
ITK
320506
402646
402647
ARHGAP28
116646
116643
224267
DLG2
364140
364135
113459

Figure S2

COMMD3 expression

All patients **Chemotherapy treated patients**

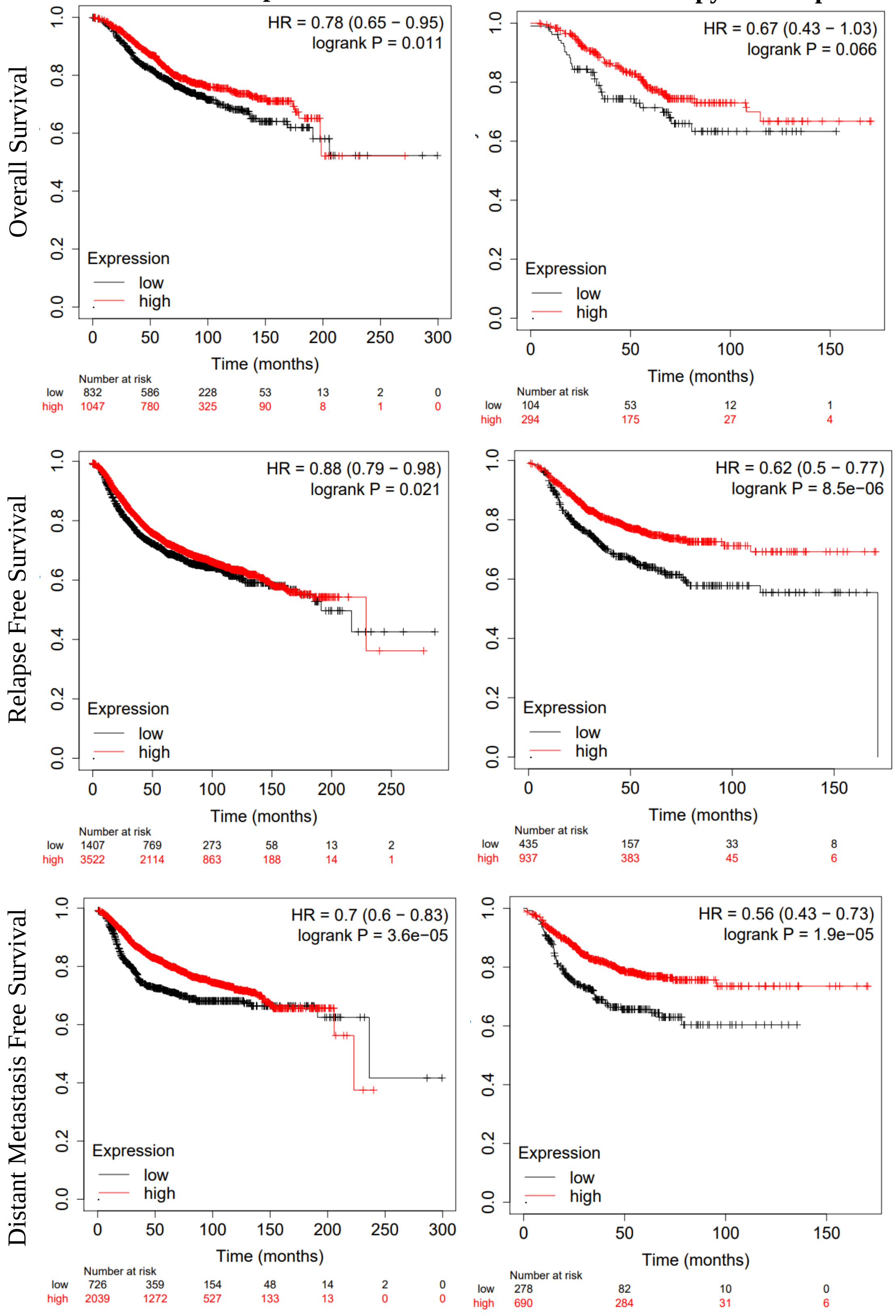

Figure S3

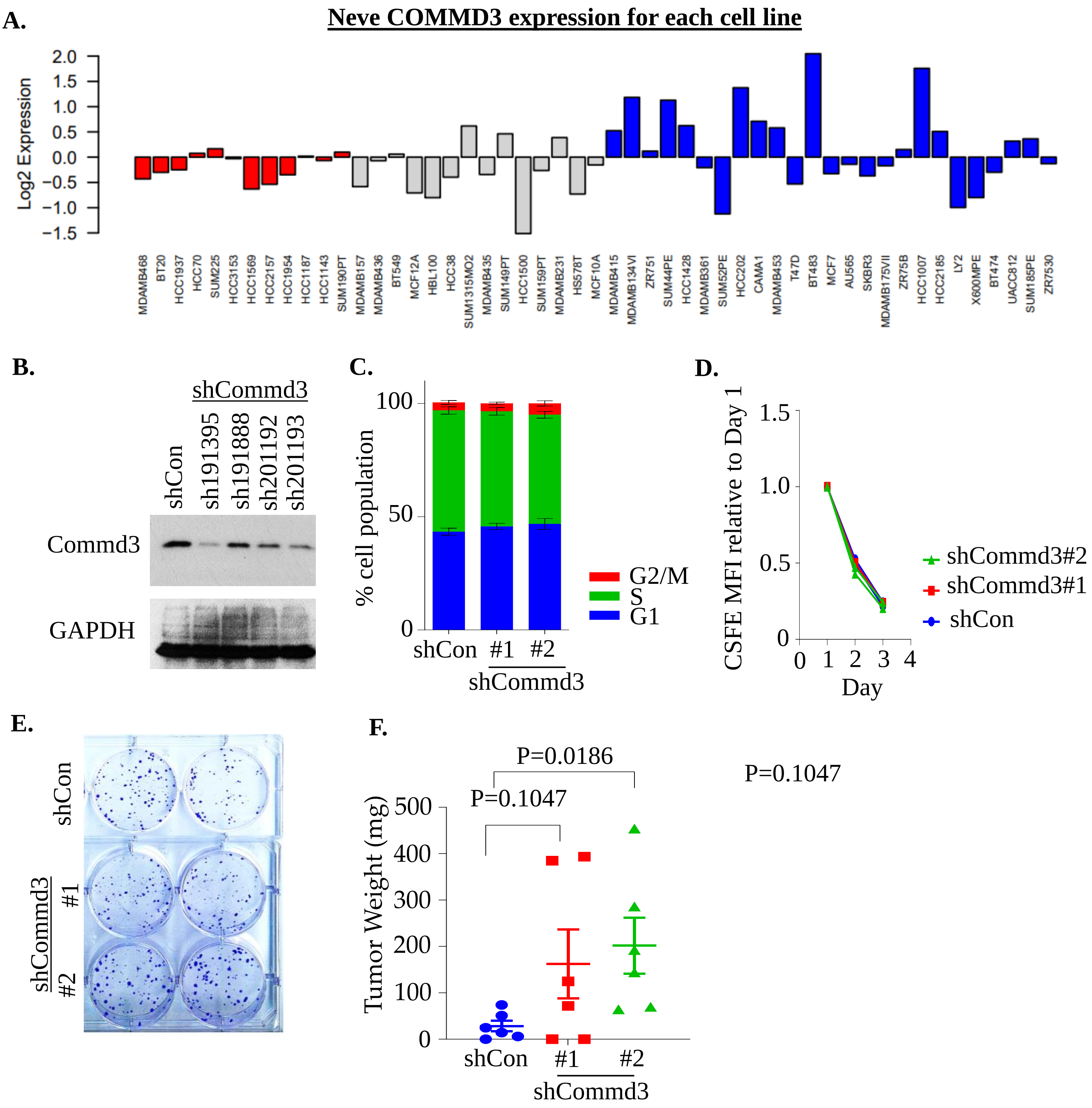
A.
Neve COMMD3 expression for each cell line
Log2 Expression
2.0
1.5
1.0
0.5
0.0
−0.5
−1.0
−1.5
MDAMB468
BT20
HCC1937
HCC70
SUM225
HCC3153
HCC1569
HCC2157
HCC1954
HCC1187
HCC1143
SUM190PT
MDAMB157
MDAMB436
BT549
MCF12A
HBL100
HCC38
SUM1315MO2
MDAMB435
SUM149PT
HCC1500
SUM159PT
MDAMB231
HS578T
MCF10A
MDAMB415
MDAMB134VI
ZR751
SUM44PE
HCC1428
MDAMB361
SUM52PE
HCC202
CAMA1
MDAMB453
T47D
BT483
MCF7
AU565
SKBR3
MDAMB175VII
ZR75B
HCC1007
HCC2185
LY2
X600MPE
BT474
UACC812
SUM185PE
ZR7530
B.
shCommd3
shCon
sh191395
sh191888
sh201192
sh201193
Commd3
GAPDH
C.
% cell population
100
50
0
shCon
#1
#2
shCommd3
G2/M
S
G1
D.
CSFE MFI relative to Day 1
1.5
1.0
0.5
0
0
1
2
3
4
Day
shCommd3#2
shCommd3#1
shCon
E.
shCon
shCommd3
#1
#2
F.
P=0.0186
P=0.1047
P=0.1047
Tumor Weight (mg)
500
400
300
200
100
0
shCon
#1
#2
shCommd3

Figure S4
